# *Dictyostelium* Nramp1, which is structurally and functionally similar to mammalian DMT1 transporter, mediates phagosomal iron efflux

**DOI:** 10.1242/jcs.173153

**Published:** 2015-09-01

**Authors:** Simona Buracco, Barbara Peracino, Raffaella Cinquetti, Elena Signoretto, Alessandra Vollero, Francesca Imperiali, Michela Castagna, Elena Bossi, Salvatore Bozzaro

**Affiliations:** 1 Department of Clinical and Biological Sciences, University of Torino, AOU S. Luigi, Orbassano 10043, Italy; 2 Department of Biotechnology and Life Sciences, University of Insubria, Via J. H. Dunant 3, Varese 21100, Italy; 3 Department of Pharmacological and Biomolecular Sciences, Università degli Studi di Milano, Via Trentacoste 2, Milano 20133, Italy

**Keywords:** DMT1, Nramp1, Nramp2, Iron homeostasis, Macropinocytosis, Bacterial infection

## Abstract

The Nramp (Slc11) protein family is widespread in bacteria and eukaryotes, and mediates transport of divalent metals across cellular membranes. The social amoeba *Dictyostelium discoideum* has two Nramp proteins. Nramp1, like its mammalian ortholog (SLC11A1), is recruited to phagosomal and macropinosomal membranes, and confers resistance to pathogenic bacteria. Nramp2 is located exclusively in the contractile vacuole membrane and controls, synergistically with Nramp1, iron homeostasis. It has long been debated whether mammalian Nramp1 mediates iron import or export from phagosomes. By selectively loading the iron-chelating fluorochrome calcein in macropinosomes, we show that *Dictyostelium* Nramp1 mediates iron efflux from macropinosomes *in vivo.* To gain insight in ion selectivity and the transport mechanism, the proteins were expressed in *Xenopus* oocytes. Using a novel assay with calcein, and electrophysiological and radiochemical assays, we show that Nramp1, similar to rat DMT1 (also known as SLC11A2), transports Fe^2+^ and manganese, not Fe^3+^ or copper. Metal ion transport is electrogenic and proton dependent. By contrast, Nramp2 transports only Fe^2+^ in a non-electrogenic and proton-independent way. These differences reflect evolutionary divergence of the prototypical Nramp2 protein sequence compared to the archetypical Nramp1 and DMT1 proteins.

## Abstract (precis)

**Summary:** The *Dictyostelium* Nramp1 transporter confers resistance to pathogenic bacteria by mediating iron efflux from phagosomes. Nramp1 and the related NrampB (formerly Nramp2) display differences in metal selectivity, electrogenicity and H^+^ dependence.

## INTRODUCTION

The Nramp (SLC11) family of metal transporters plays a major role in transition-metal ion homeostasis from bacteria to humans. In prokaryotes, three phylogenetically distinct groups of manganese transporters (MntH) are related to the Nramp proteins (Richer et al., 2003), and the crystal structure for one of them has been recently determined (Ehrnstorfer et al., 2014). Two Nramp homologs in yeast, Smf1 and Smf2, are also manganese transporters, whereas a third one, Smf3, probably transports iron from the vacuole to the cytosol (Cohen et al., 2000; Portnoy et al., 2000). Plant Nramp homologs are essential for seed germination when soil manganese or iron availability is limited (Socha and Guerinot, 2014; Cailliatte et al., 2010). The Nramp homolog in *Drosophila*, the Malvolio protein, regulates Mn^2+^ and Fe^2+^ homeostasis, affecting taste behavior (Rose et al., 2011; Folwell et al., 2006; Orgad et al., 1998). In mammals, the SLC11A family includes two genes, NRAMP1 (SLC11A1) and DMT1 (SLC11A2, formerly NRAMP2). NRAMP1 is expressed exclusively in late phagosomes of macrophages, and plays an important role in innate immunity to bacterial infection (Forbes and Gros, 2001; Blackwell et al., 2003; Nevo and Nelson, 2006; Courville et al., 2006). Polymorphic variants in or around the Nramp1 gene have been correlated with tuberculosis and leprosy (Wu et al., 2013; Johnson and Wessling-Resnick, 2012; Gruenheid et al., 1997; Supek et al., 1996; Courville et al., 2006). The paralog DMT1 is the major iron transporter at the apical membrane of intestinal cells, enabling dietary iron uptake in the duodenum. A differentially spliced DMT1 isoform, expressed at the cell surface and in endosomes, facilitates transferrin-independent iron uptake in most peripheral tissues (Montalbetti et al., 2013; Gruenheid et al., 1999; Gunshin et al., 2001; Canonne-Hergaux and Gros, 2004; Courville et al., 2006). Mutations in DMT1 have been correlated with microcytic anemia in human and rat (Bardou-Jacquet et al., 2011; Beaumont et al., 2006; Fleming et al., 1997). Altered DMT1 expression leads to iron deposition in neuronal cells and neurodegenerative disorders (Howitt et al., 2009; Urrutia et al., 2013).

Functional studies of yeast Smf1 and mammalian DMT1 in *Xenopus* oocytes have shown that both mediate proton-dependent metal ion transport (Mackenzie et al., 2006; Chen et al., 1999). DMT1 acts as an electrogenic symporter of several metals and protons, with the proton gradient providing the energy for transport (Illing et al., 2012; Mackenzie et al., 2006; Nevo and Nelson, 2006). Mammalian NRAMP1 also transport iron, manganese and copper together with protons, but the directionality of transport has long been a matter of debate, with one group of scientists favoring an antiporter mechanism, and another group supporting a symporter mechanism (Forbes and Gros, 2001; Nevo and Nelson, 2006; Courville et al., 2006).

The genome of the social amoeba *Dictyostelium discoideum* contains two Nramp genes, encoding Nramp1 and Nramp2. Similar to its mammalian ortholog, *D. discoideum* Nramp1 is recruited to phagosomes, and its inactivation enhances susceptibility to *Mycobacteria* and *Legionella* (Peracino et al., 2006, 2010). *D. discoideum* Nramp2 is instead localized in the membrane of the contractile vacuole, a tubulo-vesicular network regulating osmolarity, and contributes to iron homeostasis in synergy with Nramp1 (Peracino et al., 2013; Bozzaro et al., 2013). In contrast to most species harboring two or more Nramp homologs, *Dictyostelium* is unique in that Nramp1 and Nramp2 differ considerably in their amino acid sequence (Peracino et al., 2013). *Dictyostelium* Nramp1 belongs to the archetypical Nramp subfamily, which includes all metazoan Nramp proteins, whereas Nramp2 is closer to prototypical Nramp proteins of some aquatic protists, fungi and to the proteobacterial MntH Cα subclass (Richer et al., 2003; Cellier et al., 2007; Peracino et al., 2013). Functional studies on *Dictyostelium* Nramp1 might thus shed novel insights on the mechanism of action of the mammalian ortholog, whereas similar studies on Nramp2 might pioneer research on the role of the homologous proteins in protists, and the potential function of the contractile vacuole in divalent metal homeostasis.

Despite the divergent sequences and distinct intracellular location of Nramp1 and Nramp2, both proteins colocalize with the v-H^+^ ATPase in their respective compartments, suggesting that divalent metal transport is coupled in both cases to proton transport, as shown for other members of this family. For Nramp1, iron uptake assays with purified phagosomes suggested a vacuolar-ATPase-dependent transport (Peracino et al., 2006). Similar assays for Nramp2 are not possible owing to breakdown of the tubular contractile vacuole network following cell lysis and the difficulty of obtaining sufficiently pure and fully functional vacuole vesicles. It would be of interest, however, to know whether Nramp2 is involved in iron influx or efflux across the vacuole, and whether Nramp1 and Nramp2 differ in their metal transport specificity and regulation.

By exploiting macropinocytic uptake of the iron-sensitive fluorescent probe calcein, we show in this paper that iron is efficiently exported from *in vivo* macropinosomes of both wild-type and the Nramp2-knockout (KO), but not Nramp1-KO, mutants. We also show that both *Dictyostelium* Nramp proteins can be functionally expressed in *Xenopus laevis* oocyte plasma membrane. *Xenopus* oocytes are particularly suited for heterologous expression and characterization of transporters, as they have a very low metal ion uptake background (Chen et al., 1999). We also show for the first time that calcein can be used in this system to study iron transport with a simple and highly sensitive assay. Taken together, the results reveal that *Dictyostelium* Nramp1 is closer to mammalian Nramp1 and DMT1 than to *Dictyostelium* Nramp2 in metal ion transport properties as well as protein sequence, suggesting different origin or evolutionary divergence of the two *Dictyostelium nramp* genes.

## RESULTS

### Nramp1 mediates, and is essential for, macropinosomal iron efflux *in vivo*

**Fig. 1.**
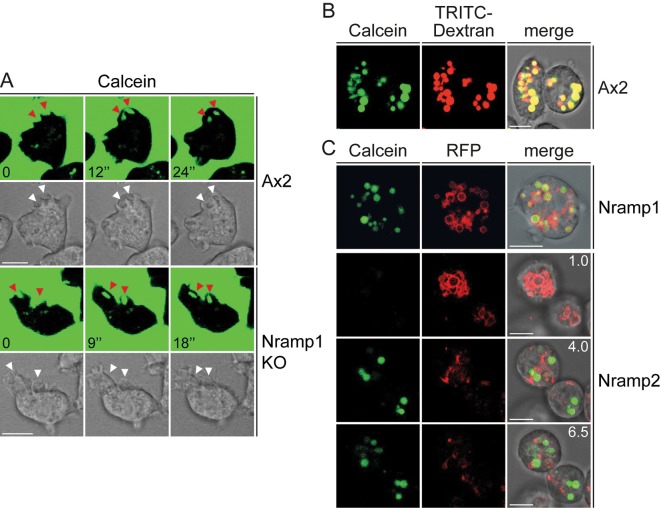
**Calcein is taken up by macropinocytosis and colocalizes with TRITC–dextran in vesicles decorated with Nramp1.** (A) Exponentially growing wild-type (Ax2) or Nramp1-KO cells were washed, resuspended in low fluorescence medium and plated on glass coverslips equipped with a Plexiglas ring. Upon addition of calcein, consecutive confocal images of single cells were taken at the indicated times (in seconds). Calcein fluorescence and corresponding phase-contrast images are shown. Arrowheads point to macropinocytic cups and vesicles. (B) AX2 wild-type cells were incubated with calcein and TRITC–dextran under shaking for 30 min, then washed and resuspended in Soerensen phosphate buffer. After plating on glass coverslips, confocal series images were taken. Coincidence of calcein and TRITC–dextran was detected in almost all vesicles, reflected in the yellow fluorescence in the merge with the phase-contrast image, indicating that calcein is taken up by macropinocytosis. (C) AX2 cells expressing Nramp1– or Nramp2–RFP were incubated with calcein under shaking for 30 min, washed and resuspended in Soerensen phosphate buffer. After plating on glass coverslips, confocal images were taken to detect calcein and the RFP fluorescence of the proteins. The merge with phase-contrast image is also shown. Calcein is found in vesicles decorated with Nramp1. In cells expressing Nramp2–RFP, calcein is not found at the bottom of the cell, where the contractile vacuole is located. In upper stacks (i.e. towards the top of the cell), calcein vesicles are evident, but they are not decorated with Nramp2, as expected given that Nramp2 is not recruited to macropinosomes. Numbers indicate the distance in µm from the bottom. Scale bars: 5 µm.

**Fig. 2.**
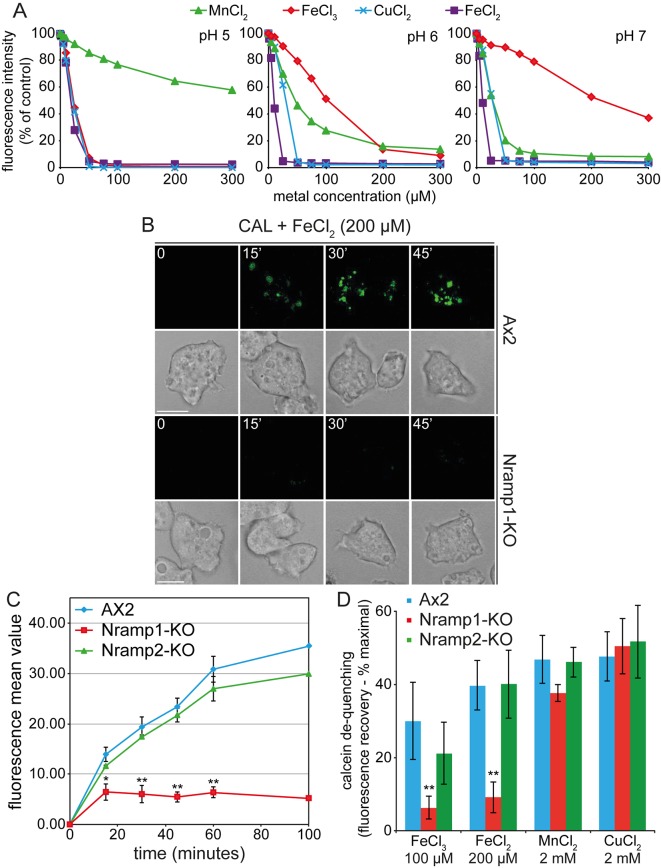
**Iron-chelated calcein is de-quenched in macropinosomes of wild-type or Nramp2-KO, but not Nramp1-KO, cells.** (A) Quantification of calcein fluorescence quenching by divalent metals. Calcein dissolved in NaOH was diluted (25 µM) in 0.1 M acetate buffer, pH 5.0 or 0.05 M PIPES or HEPES, at pH 6.0 or 7.0, respectively. The different buffers were required to maintain the right pH. Calcein was then titrated with increasing concentrations of FeCl_3_, FeCl_2_, CuCl_2_ or MnCl_2_. The fluorescence intensity (λ_ex_=485 nm, λ_em_=535 nm) was measured in non-treated white multiwell plastic plates (NUNC) in a Perkin-Elmer EnSpire Multimode Plate Reader, and the quenching expressed as a percentage of fluorescence intensity in controls containing the same amount of calcein in the absence of any divalent metal (*n*=1 experiment in duplicate). (B) Exponentially growing cells were plated on a glass coverslip and incubated with a solution of calcein (CAL) and saturating amount of Fe(Cl)_2_, which completely quenches calcein fluorescence (note the black surrounding the cells at time 0). As time progresses (shown in minutes), spots of fluorescent calcein in AX2 cells appear and increase in number and intensity, indicating iron detachment from calcein and its efflux in the cytosol. This does not occur in the Nramp1-KO mutant. (C) AX2, Nramp1-KO and Nramp2-KO cells were treated as in B but under shaking. At the times indicated, the cells were washed and their fluorescence quantified in a flow cytometer. Calcein fluorescence mean value increases steadily in AX2 and Nramp2-KO cells, but not in Nramp1-KO cells. (D) Cells were treated as in C in the presence of the indicated divalent metals, which all quench calcein fluorescence. After 30 min of incubation, cells were washed and the mean fluorescence value determined by flow cytometry. Calcein de-quenching (fluorescence recovery) was expressed relative to maximal fluorescence (i.e. to the value of calcein fluorescence in each cell type without added metal). Calcein de-quenching occurs also in Nramp1-KO cells incubated with manganese and copper, but not iron, suggesting that other transporters might be active for these metals. In the case of manganese, acidification might be sufficient to induce de-quenching (see Fig. 1). In C and D, mean±s.e.m. values of at least three independent experiments are shown. *P<0.05 or **P<0.01 (two-tailed Student's *t*-test, assuming unequal variance). Scale bars: 5 μm.

*Dictyostelium* Nramp1 has been shown to be recruited to macropinosomes and phagosomes and to facilitate iron transport in purified phagosomes (Peracino et al., 2006). To assess its activity *in vivo*, we used the metal-sensitive fluorochrome calcein for continuous monitoring of divalent metal transport. Given that vegetative cells of axenic strains, such as AX2, display intense macropinocytic activity to engulf liquid food from culture medium, we exploited the membrane impermeability of calcein to load the fluorescent probe specifically in macropinosomes, as shown by rapid uptake of the fluorochrome in macropinocytic cups and vesicles (Fig. 1A), where calcein colocalizes with TRITC–dextran, a marker of macropinosomes (Fig. 1B; Maniak, 2001). In agreement with Nramp1 recruitment in phagosomes and macropinosomes, vesicles of engulfed calcein were mostly decorated with Nramp1 in cells expressing Nramp1–RFP (Fig. 1C), whereas no colocalization was observed with Nramp2–RFP, which is found exclusively in the membrane of the contractile vacuole, a tubulo-vesicular network located at the bottom of cells adhering to a substratum (Peracino et al., 2013). As shown in Fig. 1C, this structure is visible in lower confocal stacks, whereas calcein-filled macropinosomes are evident in upper stacks, and these are totally distinct from the contractile vacuole network. Calcein fluorescence is stable at pHs between 5 and 7, but fades significantly at a pH below 4, and is selectively quenched by several divalent metal ions, but unaffected by monovalent ions (Espósito et al., 2002). This was confirmed by measuring fluorimetrically the fluorescence intensity of a calcein solution and its quenching by increasing concentrations of FeCl_3_, FeCl_2_, CuCl_2_ or MnCl_2_. Quenching was partially pH dependent. Fe^2+^ and copper efficiently quenched calcein at all pHs tested, Fe^3+^ was effective at pH 5.0 and required higher concentrations with increasing pH, whereas the opposite occurred for manganese, which was quite insensitive at pH 5.0 but quenched at pH above 6.0 (Fig. 2A).

We reasoned that upon incubating cells in growth medium with calcein and iron, or other quenching metals at saturating concentrations, macropinocytosis would lead to the uptake of the metal-quenched calcein into macropinosomes; efflux of the metals through Nramp1 (or other metal transporters) should then result in recovery of calcein fluorescence, allowing the detection of differences between Nramp1-KO mutant and the wild-type AX2 parental strain. It is worth mentioning that iron, and possibly other divalent metals, are very likely taken up by axenic lab strains only by macropinocytosis, whereas in wild-type strains living on soil and growing on bacteria the major if not unique iron source would be the bacteria, which are engulfed by phagocytosis (Bozzaro et al., 2013). When incubated with Fe^2+^-quenched calcein, fluorescent spots indicative of calcein fluorescence de-quenching were observed within 15 min in confocal images of AX2 cells, and their number and intensity increased during the subsequent 45 min. By contrast, no calcein de-quenching occurred in Nramp1-KO cells (Fig. 2B). Flow cytometry assays confirmed the qualitative data: compared to control cells incubated with calcein alone, calcein de-quenching (i.e. fluorescence recovery) was minimal in the Nramp1-KO mutant and did not increase over time, whereas it increased steadily in AX2 and Nramp2-KO cells (Fig. 2C). Remarkably, incubating cells with calcein and either FeCl_2_ or FeCl_3_ did not affect the outcome: both were exported from macropinosomes in AX2 and Nramp2-KO, but not in Nramp1-KO, mutant cells (Fig. 2D). Even when cells were incubated with calcein at non-saturating iron concentrations, fluorescence recovery was still significantly lower in Nramp1-KO, and Nramp1 and Nramp2 double-KO mutants than in Nramp2-KO or AX2 control cells (supplementary material Fig. S1). The possibility that calcein is not dequenched in Nramp1-KO mutant due to a stronger (below pH 4.0) and prolonged acidification of the macropinosomes in this mutant can be excluded because no differences in the extent and timing of acidification are found between the mutant and the parental AX2 strain (data not shown). In addition, no significant differences between AX2, Nramp1- or Nramp2-KO cells were found when Mn^2+^ or Cu^2+^ were tested in place of iron, even at a tenfold higher concentration (Fig. 2D). Thus we conclude that Nramp1 is required for iron efflux from macropinosomes *in vivo*, whereas other transporters in place of Nramp1 mediate transport of copper and possibly manganese. Concerning the latter, however, it cannot be excluded that the apparent calcein fluorescence recovery might be solely due to macropinosome acidification leading to detachment of manganese from calcein (see Fig. 2A).

### Fe^2+^, but not Fe^3+^, is transported in *Xenopus* oocytes expressing *Dictyostelium* Nramp1 or Nramp2

The previous experiments have shown that Nramp1 is essential for iron export from macropinosomes and that both Fe^3+^ and Fe^2+^ are *prima facie* exported from macropinosomes. To better characterize the iron transport properties of *Dictyostelium* Nramp1 or Nramp2, we expressed the proteins in the membrane of *Xenopus* oocytes. Injecting unmodified cRNAs resulted in a very low level of functional expression, which was not surprising as both Nramp1 and Nramp2 are not plasma membrane proteins. To increase expression in the plasma membrane, the N and C-termini of both proteins were replaced with the N- and C-termini of rat DMT1, according to the scheme in supplementary material Fig. S2. Rat DMT1 (rDMT1), which has been successfully expressed in *Xenopus* oocytes (Gunshin et al., 1997; Sacher et al., 2001; Cohen et al., 2003; Mackenzie et al., 2006), was used as internal control. The chimeric Nramp1 and Nramp2 containing the short rDMT1 termini were named c-Nramp1 and c-Nramp2.

**Fig. 3.**
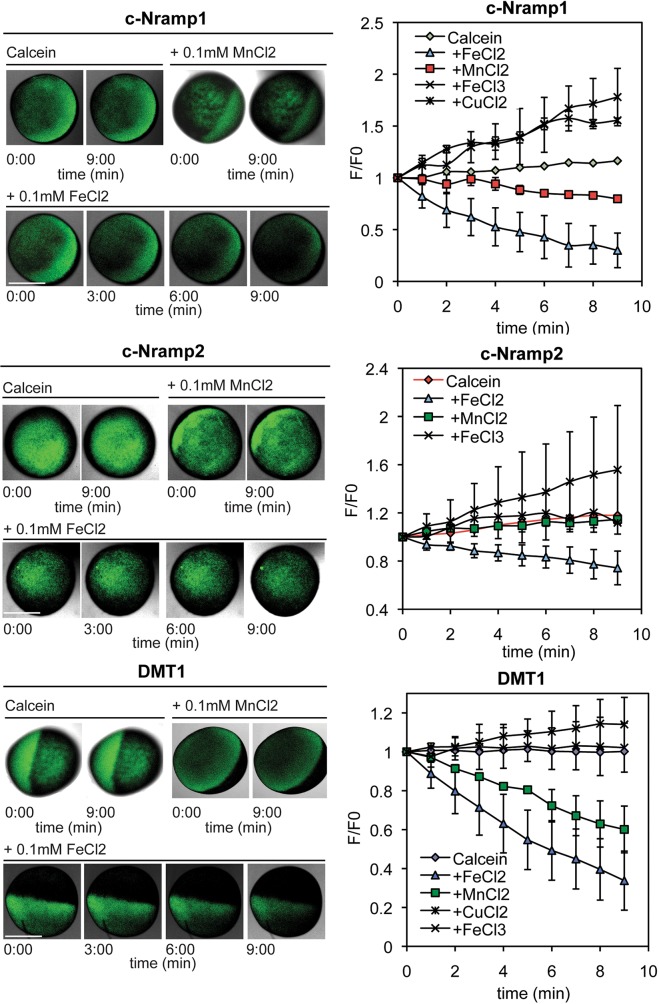
**Live imaging of divalent metal transport in *Xenopus* oocytes using calcein fluorescence.** *Xenopus* oocytes expressing c-Nramp1, c-Nramp2 or rDMT1 were injected with calcein and perfused with a solution containing or not the indicated metal. Confocal series images were taken at the time indicated. Left, in the absence of any metal, calcein fluorescence was stable in all oocytes for all the length of the experiment. In the presence of Mn(Cl)_2_ or Fe(Cl)_2_ fluorescence decreased within minutes, although this was less evident for c-Nramp2-expressing oocytes. Right, the fluorescence changes over time were quantified by measuring the ratio of fluorescence intensity at the time indicated (F) versus fluorescence at time 0 (F0). Mean±s.d. are shown for each group of oocytes [c-Nramp1 *n* values are: Mn, 3; Fe(II), 8; Fe(III), 4; Cu, 5; c-Nramp2 *n* values are: Mn, 2; Fe(II), 7; Fe(III), 11; Cu, 5; rDMT1 *n* values are: Mn, 2; Fe(II), 8; Fe(III), 8; Cu, 7]. Statistical analysis was undertaken at the 9-min time point using a two-tailed Student's *t*-test, assuming unequal variance: Fe(II) versus control was *P*<0.05 in all cases; Cu versus control was *P*<0.05 for c-Nramp1. Scale bars: 0.5 mm.

We introduced a novel assay based on metal-induced changes in calcein fluorescence. Oocytes transfected with cRNA coding for c-Nramp1, c-Nramp2 or rDMT1 were microinjected with calcein, incubated with divalent metals, and the dynamics of fluorescence quenching was checked by confocal microscopy. In the absence of external metals, calcein fluorescence in control oocytes or oocytes expressing the transporters was basically unaltered (Fig. 3). In the presence of metals (manganese, iron or cobalt), the fluorescence ratio F:F0 in control oocytes oscillated within the range 1–1.15 for all the length of the experiment (data not shown). In oocytes expressing c-Nramp1, c-Nramp2 or rDMT1 and incubated with FeCl_2_, the fluorescence intensity decreased rapidly, as shown in confocal images of representative oocytes and in the quantitative data of Fig. 3. Fe^2+^ strongly quenched calcein fluorescence in c-Nramp1- and rDMT1-expressing oocytes, with an ∼50% reduction of F:F0 after 3 and 6 min of incubation for c-Nramp1 or DMT1, respectively. In c-Nramp2-expressing oocytes Fe^2+^ led to fluorescence quenching but at a lower rate. Mn^2+^ also lowered calcein fluorescence in c-Nramp1- and rDMT1-, but was ineffective in c-Nramp2-expressing oocytes (Fig. 3). Interestingly, when incubated with Fe^3+^ or Cu^2+^, calcein fluorescence did not decrease, but increased linearly in c-Nramp1-expressing oocytes, reaching an F:F0 value of 1.5 after 6 min (Fig. 3). In some experiments, calcein was co-injected with Fe^2+^ to further reduce calcein fluorescence, and the oocytes were then incubated with FeCl_3_. In addition, in this case Fe^3+^ led to increased calcein fluorescence (data not shown). A similar effect was observed for Fe^3+^, but not for Cu^2+^, in c-Nramp2-expressing oocytes, but the results were highly variable from experiment to experiment. In oocytes transfected with rDMT1, Fe^3+^ also led to a slight increase in F:F0, which was, however, not significantly different from non-injected eggs. Thus, it appears that *Dictyostelium* Nramp1, like rDMT1, transports Fe^2+^ and Mn^2+^, but not Fe^3+^ or Cu^2+^, whereas it is the latter two ions that interact, at least with Nramp1, in a way that favors release of calcein-quenching metals from the cytoplasm, thus increasing calcein fluorescence. Iron and copper, in particular, are enriched in the animal pole of *Xenopus* oocytes (Popescu et al., 2007). c-Nramp2 differs from c-Nramp1 and rDMT1 in that only Fe^2+^ is transported, though at a reduced rate, and only Fe^3+^ leads to increase in calcein fluorescence.

### Nramp1-mediated, but not Nramp2-mediated, metal transport elicits pH-dependent electrogenic currents

**Fig. 4.**
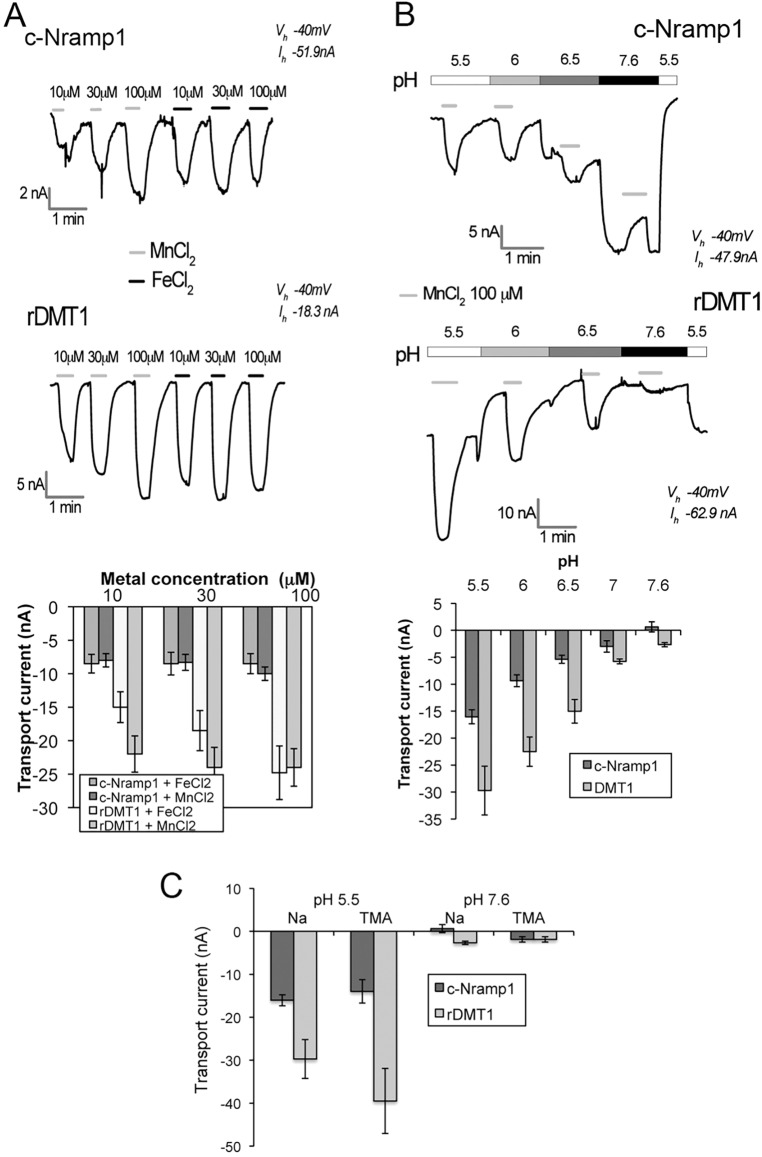
**Fe and Mn elicit pH-dependent transport currents in oocytes expressing c-Nramp1 or rDMT1.** (A,B) Oocytes expressing c-Nramp1 or rDMT1 were clamped at a constant voltage of −40 mV in sodium solution at (A) pH 6.0 or (B) at different pH, and perfused with the indicated metal. Currents were recorded as described in the Materials and Methods. Effects of increasing metal ion concentrations and pH on transport currents for a single oocyte are shown on top and the mean±s.e.m. values for 8 to 20 oocytes for each condition in three different experiments are shown at the bottom of the figure. *P*<0.05 for c-Nramp1 and rDMT1 compared to control oocytes or at pH 5.5 versus pH 7.6. Both Mn and Fe evoked currents mediated by c-Nramp1 and rDMT1, which are maximal at pH 5.5, smaller at higher pH and switch outwardly at basic pH, at least for c-Nramp1. (C) Oocytes were perfused with Mn(Cl)_2_ in sodium or TMA solution. The outward current evident with c-Nramp1 disappears if Na is replaced by TMA, suggesting the presence of a Na leakage current that is blocked by the presence of the co-transported ion (Mn). For all the traces recorded, the holding current (*I*_h_) is indicated. Mean±s.e.m. values of 5 to 10 experiments with 10 to 50 oocytes are shown. *P*<0.05 for c-Nramp1 and DMT1 compared to control oocytes or at pH 7.6.

To further characterize the *Dictyostelium* transporters, the oocytes were tested electrophysiologically to verify the presence of transport currents associated with metal transport. First, the c-Nramp1-expressing oocytes were clamped at a constant voltage of −40 mV in a sodium solution at pH 6 and perfused with 0.01 to 0.1 mM FeCl_2_ or MnCl_2_. At all concentrations of substrates tested, c-Nramp1 elicited inward currents of ∼9 nA compared to ∼20 nA in rDMT1 oocytes (Fig. 4A)*.* The similarity of the currents elicited by FeCl_2_ or MnCl_2_ led us to use the latter, being more stable, for testing the pH dependence of the currents. As shown in Fig. 4B, in c-Nramp1- and rDMT1-expressing oocytes the currents became smaller with increasing pH, apparently switching to being outwardly at pH 7.6 only in the case of c-Nramp1. This latter change did not occur in tetramethylammonium chloride (TMA) solution at the same pH, where the currents were similar in c-Nramp1 and rDMT1 (Fig. 4C). The behavior of c-Nramp1 resembles that of the yeast Smf1p transporter expressed in *Xenopus* oocytes (Sacher et al., 2001; Chen et al., 1999), and can be explained by divalent ions blocking a Na^+^ slippage current, as proposed previously (Nelson et al., 2002). Further investigation at pH 7.6 and in the absence of divalent metal ions confirmed that sodium or lithium elicited a large slippage current in c-Nramp1, but not in rDMT1, which could be partially blocked by lowering the pH (supplementary material Fig. S3).

**Fig. 5.**
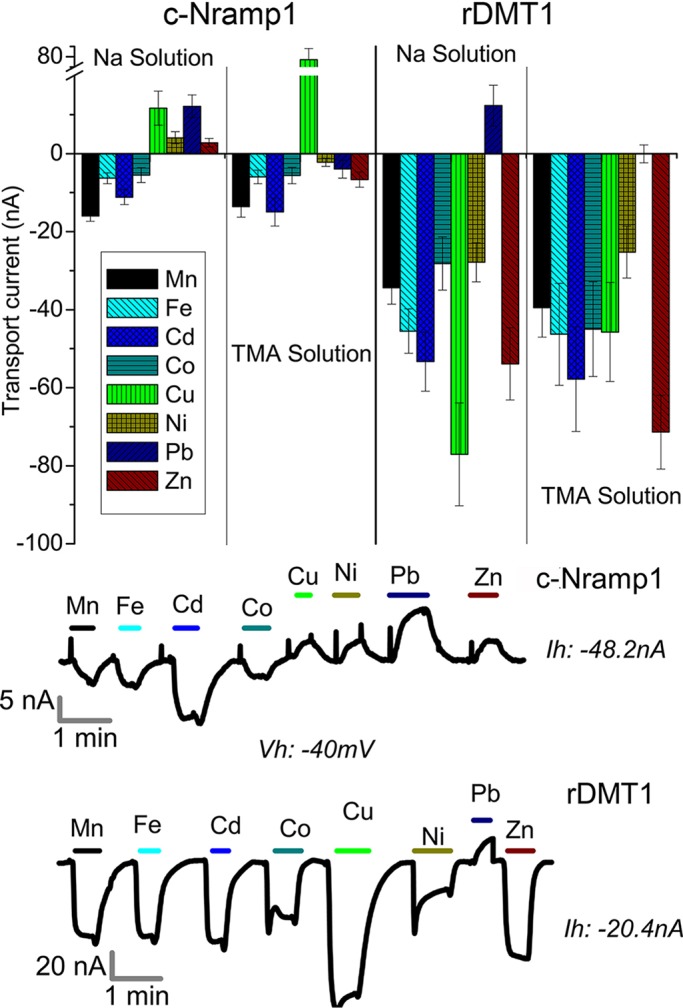
**Differential effects on transport currents by metal ions in *Xenopus* oocytes expressing c-Nramp1 or rDMT1.** Oocytes expressing c-Nramp1 or rDMT1 were clamped at a constant voltage of −40 mV and perfused with the indicated metal at a concentration of 0.1 mM in either sodium or TMA solution at pH 6.0. Currents were recorded as described in the Materials and Methods. Top, mean±s.e.m. values of a variable number of experiments for each condition, with four oocytes in three experiments and up to 50 oocytes in 10 experiments, and, bottom, recording of single oocytes in TMA. For all the traces recorded, the holding current (*I*_h_) was reported.

The largest transport current (i.e. maximal functionality), in the absence of sodium is recorded for c-Nramp1 at pH 5.5, similar to rDMT1 (Fig. 4C). Under these conditions, 0.1 mM Fe^2+^, Mn^2+^, Cd^2+^ or Co^2+^ elicited inward currents, and Ni^2+^, Pb^2+^ and Zn^2+^ elicited small inward or outward currents in TMA or sodium solution, respectively (Fig. 5). This discrepancy can be explained by the presence of the already mentioned Na^+^-slippage current, that is present also at pH 5.5 (supplementary material Fig. S3). Cu^2+^ induced outward currents in both sodium and TMA solutions (Fig. 5), confirming that it is not transported by Nramp1 but interacts and alters the transporter in some way. In rDMT1-expressing oocytes, the transport currents elicited by the divalent metals tested were larger, particularly by Cu^2+^, and only Pb^2+^ induced an outward or no current in Na^+^ or TMA solution, respectively (Fig. 5). In contrast to c-Nramp1 and DMT1, c-Nramp2 did not elicit any measurable transport current, either in sodium or in TMA solution, irrespective of the different holding potential tested (−100 mV, 0 mV or +20 mM), the pH of the bath solution, or the presence of Fe^2+^ ion outside or inside the oocyte (data not shown).

### pH-dependence and specificity of iron transport by Nramp1 are confirmed by radiochemical uptake assay

**Fig. 6.**
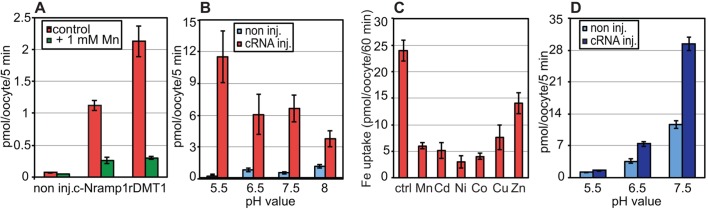
**Radiochemical uptake assays with *Xenopus* oocytes expressing c-Nramp1, c-Nramp2 or rDMT1.** (A) Control oocytes (non inj.) or oocytes injected with cRNA for c-Nramp1 or rDMT1 (cRNA inj.) were incubated with 0.1 mM ^55^Fe(Cl)_2_ in the presence or not of 1 mM Mn(Cl)_2_ as described in the Materials and Methods. At the end of incubation, the oocytes were washed and dissolved in SDS, and the accumulated radioactivity was counted in a liquid scintillation counter. Mean±s.e.m. values for groups of 8–10 oocytes, in a representative experiment of three independent, are shown. Both c-Nramp1 and rDMT1 mediate iron uptake, which is inhibited by Mn. *P*<0.001 for Nramp1 control versus non inj. Control; *P*<0.003 for Nramp1 Mn versus Nramp1 control (Student's *t*-test). (B,C) Oocytes injected or not with c-Nramp1 were assayed for transport of ^55^Fe(Cl)_2_ (B) at different pH or (C) in the presence of different metal ions. In B, mean±s.e.m. values for groups of 8–10 oocytes in a representative experiment of three independent are shown. *P*<0.05 for cRNA inj. pH 5.5 versus cRNA inj. pH 6.5; *P*<0.004 for cRNA inj. pH 5.5 versus cRNA inj. pH 8 (Student's *t*-test). In C, data represent ^55^Fe uptake in the absence (control) or presence of the indicated divalent metal at a concentration of 1 mM. Data are the mean±s.e.m. for groups of 8–10 oocytes in three separate experiments. *P*<0.04 for each metal versus control (Student's *t*-test). Uptake by c-Nramp1 is pH dependent, being maximal at pH 5.5, and inhibited by all metals tested, although to a lower extent for zinc. (D) Oocytes injected with c-Nramp2 were tested for transport of 0.1 mM ^55^Fe(Cl)_2_ at the pH values indicated. Mean±s.e.m. values for groups of 8–10 oocytes in a representative experiment of three independent are shown. *P*<0.001 for cRNA inj. pH 5.5 versus cRNA inj. pH 7.5; c-Nramp2 induces significant iron accumulation only at pH 7.5 (Student's *t*-test).

*Xenopus* oocytes were also tested for uptake of radiolabeled iron in the presence of ascorbic acid to maintain iron in the reduced form. Following incubation with 0.1 mM ^55^FeCl_2_, the oocytes were washed and lysed to measure intracellular radioactivity. Compared with control oocytes, a significant level of radioactivity was detected in c-Nramp1- or rDMT1-injected oocytes, which was inhibited in both cases by Mn^2+^ addition (Fig. 6A). Absorption of radiolabeled iron was maximal at acidic pH (Fig. 6B), and inhibited not only by Mn^2+^, but also by Cd^2+^, Co^2+^, Ni^2+^, Cu^2+^ and, to a lesser extent, Zn^2+^ (Fig. 6C). In the case of c-Nramp2, a low, though still significant, uptake of radiolabeled iron, was detected at pH 6.5, with a slight increase at pH 7.5 (Fig. 6D). At pH 5.5, no difference could be detected compared with control oocytes.

## DISCUSSION

We have shown for the first time that calcein can be successfully used to study iron transport in macropinosomes as well as in *Xenopus* oocytes. Calcein, a metallosensor consisting of fluorescein conjugated to an EDTA-like moiety, has been mostly used in the form of the membrane-permeant non-fluorescent precursor calcein-acetoxymethyl ester (CAL-AM), to detect changes in cytosolic or mitochondria labile iron pool, following intracellular cleavage of the ester bond (Espósito et al., 2002; Kakhlon and Cabantchik, 2002; Petrat et al., 2002; Picard et al., 2000). In this paper we exploited, instead, the membrane impermeability of calcein and its uptake by macropinocytosis. By incubating cells with metal-chelated, thus non-fluorescent, calcein, fluorescence de-quenching in macropinosomes can be detected following metal release and efflux to the cytosol. We have shown that in the case of iron-chelated calcein, fluorescence de-quenching occurred in macropinosomes of wild-type or Nramp2-KO cells, but not in the Nramp1-KO mutant, indicating that Nramp1 is essential for, and mediates, iron efflux from macropinosomes. In *Dictyostelium*, macropinocytosis and phagocytosis are differentially regulated, but macropinosomes and phagosomes, once formed, share the same intracellular traffic pathway, characterized by rapid recruitment in their membrane of Nramp1 and v-H^+^ ATPase and gradual import of lysosomal enzymes (Peracino et al., 2006, 2010; Gotthardt et al., 2006). We can therefore assume that the Nramp1-mediated iron efflux observed in macropinosomes occurs also in phagosomes.

The finding that iron is exported from macropinosomes through Nramp1 adds an important piece of evidence concerning the mechanism of action of this protein and its mammalian ortholog. It is generally accepted that NRAMP1 is a proton-dependent iron transporter, but the directionality of transport has long been a matter of debate, with one hypothesis favoring proton-driven iron release from the phagosomes, thus starving the engulfed pathogens of an important element (Forbes and Gros, 2001; Nevo and Nelson, 2006; Courville et al., 2006), and the alternative hypothesis favoring accumulation of iron in the phagosomal lumen with proton exchange, thus leading to formation of toxic oxygen radicals (Goswami et al., 2001; Techau et al., 2007). By using purified phagosomes of *Dictyostelium* cells, we have previously shown that Nramp1 was essential for radiolabeled iron transport, which was imported into the phagosomes at a slightly acidic pH if a source of ATP was provided to activate the v-H^+^-ATPase (Peracino et al., 2006). By using iron-chelated calcein, we have now shown in this paper that Nramp1 is also essential *in vivo*, and that it mediates iron efflux from macropinosomes to the cytosol, thus strongly supporting the notion that Nramp1 favors iron depletion from phagosomes.

Phagocytosis is exploited by *Dictyostelium* cells to engulf bacteria that are the major, if not unique, source of food in the wild, and as such a potential entry for pathogenic bacteria (Bozzaro, 2013; Bozzaro et al., 2008). A few bacteria species, such as *Legionella pneumophila*, are taken up by macropinocytosis rather than phagocytosis (Peracino et al., 2010). *Dictyostelium* Nramp1-KO mutants are more susceptible to infection by both Mycobacteria and Legionella, irrespective of their different uptake mechanism, but in agreement with the common function of Nramp1 in phagosomes and macropinosomes. *Dictyostelium* Nramp1 displays, therefore, both a nutritive and a protective function, favoring cytosolic accumulation of an essential element upon bacterial digestion and simultaneously starving pathogenic bacteria in phagosomes or macropinosomes from iron. Such a dual role is similar to that of the Nramp1 ortholog in macrophages, which recycles iron from ingested erythrocytes, while starving ingested pathogens from iron or manganese (Cellier, 2012; Cellier et al., 2007).

Remarkably, Nramp1-dependent calcein de-quenching was observed in macropinosomes of cells upon incubation with both Fe^2+^- or Fe^3+^-quenched calcein. In the experiments with *Xenopus* oocytes, we have shown, however, that only Fe^2+^ is transported by Nramp1. This suggests that *Dictyostelium* cells are able to reduce Fe^3+^ in macropinosomes or phagosomes. In macrophages, phagosomal or endosomal Fe^3+^ reduction is mediated by ferrireductases of the STEAP family (Ohgami et al., 2006). No STEAP homologs exist in the *Dictyostelium* genome, which harbors, however, three putative ferric reductase genes (Bozzaro et al., 2013) that could take over this function if they are recruited to the macropinosomal and phagosomal lumen. Preliminary experiments in our laboratory have shown that at least one of these ferric reductases is indeed recruited to phagosomes (B.P., unpublished data).

For better characterization of the transport properties of both Nramp1 and Nramp2, we expressed the proteins in the plasma membrane of *Xenopus* oocytes. In addition to well-established radiochemical uptake assays and electrophysiological recording, we have developed a novel assay based on calcein injection into the oocyte and potential quenching of the fluorescence by imported divalent metals. We have shown that calcein fluorescence is stable in control oocytes, but is quenched within a few minutes by Fe^2+^ in oocytes expressing chimeric Nramp1 and Nramp2 or rDMT1, a process that can be easily visualized by confocal microscopy and can be quantified. The calcein-quenching rate by Fe^2+^ and Mn^2+^ was roughly comparable in c-Nramp1- and rDMT1-expressing oocytes, suggesting a similar transport rate. It was instead reduced for Fe^2+^ and undetectable for Mn^2+^ in c-Nramp2 expressing oocytes. An unexpected interesting result was that Fe^3+^ and Cu^2+^ increased rather than decreased calcein fluorescence in c-Nramp1 expressing oocytes. These cations also failed to quench calcein in Nramp2- and rDMT1-expressing oocytes, suggesting that these metals are not imported, but nevertheless interact with the transporters, very likely favoring efflux of calcein-quenching metals from the oocyte. Interestingly, metal ion binding studies in crystals of the homolog ScaDMT have recently shown that Cu^2+^ binds to the same location as Mn^2+^ and Fe^2+^, but at a slightly shifted position (Ehrnstorfer et al., 2014). This could explain why copper, and presumably also Fe^3+^, are not transported, but could possibly make the channel leaky.

To our knowledge, only one paper has been published so far on the characterization of metal transporters in *Xenopus* oocytes with fluorescent probes, namely for investigating DMT1 transport using Phen Green SK (Illing et al., 2012). Those authors showed that DMT1 transports Fe^2+^ and Mn^2+^, but not Fe^3+^, Cu^2+^, Cr^2+^ or Cr^3+^ among others. These results are in agreement with the present results with calcein for rDMT1.

The radiochemical and electrophysiological analyses confirm that *Dictyostelium* Nramp1 is a proton-dependent Fe^2+^ transporter that shares with mammalian DMT1 several features: both transport Fe^2+^ and Mn^2+^, in both cases transport is maximal at acidic pH, quite absent at neutral or basic pH, and is electrogenic, with the size of inward currents also depending on pH. Nramp1 differs from rDMT1 for a large Na^+^ or H^+^ slippage.

In so far as *Dictyostelium* Nramp1 is the ortholog of mammalian NRAMP1, and both proteins are recruited to phagosomes in *Dictyostelium* cells as well as macrophages, the present results are clear-cut evidence in favor of the idea that these proteins mediate proton-driven iron efflux from these vesicles (Forbes and Gros, 2001; Nevo and Nelson, 2006; Soe-Lin et al., 2010), challenging the hypothesis that they act as antiporters (Goswami et al., 2001; Techau et al., 2007).

**Fig. 7.**
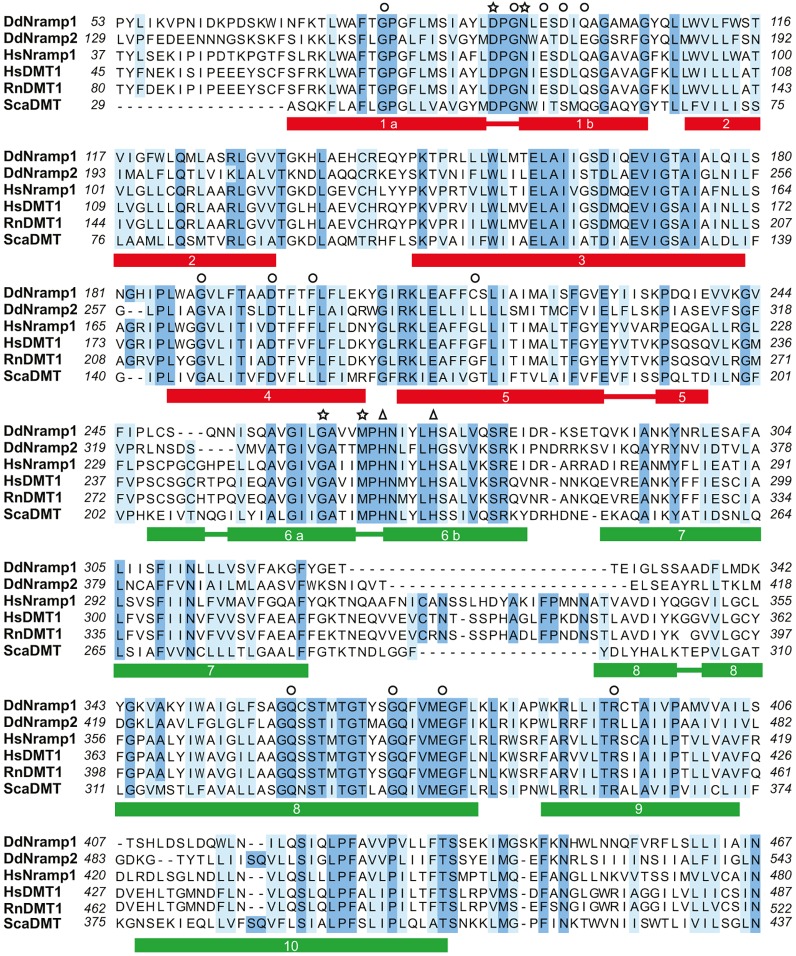
**Sequence alignment.** Sequences of *Dictyostelium* Nramp1 and Nramp2, human Nramp1 and DMT1 (formerly Nramp2) (isoform1), rat DMT1 and *Staphylococcus capitis* DMT were aligned with ClustalW. Identical or homologous residues are shown in sky blue or light blue, respectively, and non-conserved residues are in white. Only the first ten transmembrane domains are shown, with the numberings reporting the amino acid residues of each protein. The predicted transmembrane domains are as described previously (Czachorowski et al., 2009; Ehrnstorfer et al., 2014). The stars point to the four amino acid residues coordinating iron binding, and the triangles to the two histidine residues required for proton dependency of transport (Czachorowski et al., 2009; Ehrnstorfer et al., 2014). Amino acids whose spontaneous or induced mutations in human or rodent DMT1 result in reduction of uptake and/or changes in specificity or pH dependency are indicated by circles (see: Fleming et al., 1997; Lam-Yuk-Tseung et al., 2003; Cohen et al., 2003; Courville et al., 2006; Beaumont et al., 2006; Czachorowski et al., 2009; Iolascon et al., 2006).

*Dictyostelium* Nramp2 expressed in *Xenopus* oocytes differs from Nramp1 and DMT1 – it has a lower rate of Fe^2+^ transport, undetectable Mn transport, independence from acidic pH, and very low, if not absent, charge movements. To what extent these differences are due to less-efficient Nramp2 expression in the oocyte membrane or to structural differences between Nramp2 and Nramp1 remains open. Sequence comparison of *Dictyostelium* Nramp1 and Nramp2 with mammalian Nramp1 and DMT1 reveals a high degree of conservation, yet with some interesting differences, which can be highlighted by comparing the proteins with the recently determined first crystal structure of a Slc11 (Nramp) transporter, the prokaryotic ScaDMT (Ehrnstorfer et al., 2014). Crystallography and functional topology studies (Czachorowski et al., 2009) show that two symmetrically oriented helics in the center of the membrane bilayer, formed by the second half of the first transmembrane α-helix are face-to-face with a symmetric half of the sixth α-helix, give rise to the ion channel and to the substrate-binding site. Four residues in both helices coordinate Mn^2+^, Fe^2+^ and Cd^2+^, but not Ca^2+^. These residues, as well as two histidine residues, presumably required for proton co-transport, are conserved in ScaDMT and mammalian NRAMP proteins as well as *Dictyostelium* Nramp1 and Nramp2 (Fig. 7). Of 15 amino acid residues whose mutations in human or murine DMT1 have been shown to affect uptake, substrate specificity, pH dependence of transport or leak current (Cohen et al., 2003; Lam-Yuk-Tseung et al., 2003; Courville et al., 2006; Nevo and Nelson, 2006; Bardou-Jacquet et al., 2011; Beaumont et al., 2006; Fleming et al., 1997), only one is modified in *Dictyostelium* Nramp1 (with an invariant C116 compared to G208 in HsDMT1), whereas three are significantly different in Nramp2 (A171, E175 and L290 corresponding to E87, Q91, G208 in HsDMT1) (supplementary material Fig. S4). Interestingly, the equivalent mutations E87A, Q91D and G208R in rat DMT1 result in decreased metal uptake in *Xenopus* oocytes (Nevo and Nelson, 2006), and can thus explain the lower iron and manganese uptake reported in this paper for c-Nramp2.

Remarkably, all these different amino acid residues of *Dictyostelium* Nramp2 are conserved in Nramp2 homologs of the other four Dictyostelid species sequenced so far (data not shown; Basu et al., 2013). Thus, they are suggestive of basic different properties between Dictyostelid Nramp1 and Nramp2. In general, Nramp1 and Nramp2 are useful tools for deciphering the mechanism of action of archetypical (Nramp1) versus prototypical (Nramp2) proteins. Archetypical Nramp proteins are found, not only in Dictyostelids, but also in all higher eucaryotes from *Arabidopsis* to mammals, whereas prototypical Nramp proteins are found, in addition to Dictyostelids, in protists, such as *Chlamydomonas* and *Volvox*, some fungi and in a subgroup of proteobacteria (Richer et al., 2003). The Dictyostelids are the only species known so far, which harbor one archetypical and one prototypical protein (Richer et al., 2003; Peracino et al., 2013). Given that *Dictyostelium discoideum* Nramp2 is a prototypical Nramp protein and is not the ortholog of mammalian DMT1 (formerly Nramp2), to avoid confusion the protein (and the gene) will be re-named from now on as NrampB (formerly Nramp2). This change is effective in the organism database (see www.dictybase.org).

In contrast to Nramp1, which is recruited to phago-lysosomes, both in *Dictyostelium* cells and in macrophages, *Dictyostelium* NrampB is localized in the membrane of the contractile vacuole network, which is also highly enriched with the v-H^+^-ATPase (Heuser et al., 1993). Aside from its regulatory activity in water and Ca^2+^ sequestration, little is known on the evolutionary origin and function of this complex structure, which is present in the social amoebae and in fresh-water protists, but absent in other eukaryotes (Plattner, 2013). Although close homologs of Dictyostelid NrampB are present in the genomes of *Chlamydomonas* and *Volvox*, whether the proteins are localized in their contractile vacuoles as in *Dictyostelium* is not known. We have suggested that the contractile vacuole can act as a reservoir or sink for excess labile iron and other divalent metals, which can be excreted, upon vacuole fusion with the plasma membrane, or mobilized back to the cytosol, depending on cell need (Bozzaro et al., 2013). Future site-directed mutagenesis studies will help to elucidate the mechanism of action of NrampB and shed insights on both iron transport across the contractile vacuole membrane and the function of the contractile vacuole in divalent metal homeostasis.

## MATERIALS AND METHODS

### *Xenopus* oocyte solutions

The oocyte (ND96 and MBS) solutions had the following composition (in mM), ND96: NaCl 96, KCl 2, MgCl_2_ 1, CaCl_2_ 1.8, HEPES 5, pH 7.6; MBS: NaCl 88, KCl 1, NaHCO_3_ 2.4, HEPES 15, pH 7.6, Ca(NO3)_2_ 0.30, CaCl_2_ 0.41, MgSO_4_ 0.82, penicillin 10 µg/ml, streptomycin sulfate 10 µg/ml, gentamycin sulfate 100 µg/ml, nystatin 10 U/ml. The external control solution contained in mM: NaCl or TMACl, 98; MgCl_2_, 1; CaCl_2_, 1.8, HEPES or MES 5. The final pH values of 6.0, 5.5 or 7.5 were adjusted with HCl and NaOH (Bossi et al., 2007).

### *Dictyostelium* cell cultures

The *Dictyostelium discoideum* parental strain AX2 was used. Single and double Nramp1- and Nramp2-KO mutants or cells expressing Nramp1– or Nramp2–GFP were generated previously and are as described by Peracino et al. (Peracino et al., 2006, 2013). For generating RFP-fused Nramp1 and Nramp2 proteins, the corresponding genes were cloned in the EcoRI site of the 389-2 vector (C-terminal mRFPmars) (Fischer et al., 2004). All strains were cultured axenically in AX2 medium (Watts and Ashworth, 1970) in Erlenmeyer flasks under shaking at 150 rpm and 22°C on a gyratory shaker in a climatic cabinet (Kuehner, Basel, Switzerland) as described previously (Bussolino et al., 1991). Blasticidin at a concentration of 0.01 mg/ml was added to the knockout mutants. Cells expressing GFP-fused proteins were cultured in the presence of 0.01 to 0.03 mg/ml G418.

### Preparation of chimeric cDNA and cRNA and expression in *Xenopus* oocytes

Chimeric cDNAs and RNAs were prepared as described previously (Soragna et al., 2004). Briefly, for generating cDNAs, the restriction sites Eco47III at the N-terminus (position +99 and +290 for *nramp1* and *nramp2*, respectively) and HpaI at the C-terminus (position +1620 and +1750 for *nramp1* and *nramp2*, respectively) were inserted by PCR amplification in cDNA coding for the proteins in pGEMT. Rat *dmt1* (NP037305.2) in pSport was mutagenized by site-directed mutagenesis with overlapping primers to insert the Eco47III site (at position +171) and the NsiI site (at position +1725) (Forlani et al., 2001). The plasmids were digested and joined according to the scheme shown in supplementary material Fig. S2. The chimeric constructs were fully sequenced before mRNA transcription.

For mRNA transfection, the cDNA encoding the desired protein cloned into the pSPORT-1 vector was linearized with NotI, and cRNA synthesized *in vitro* in the presence of Cap Analog and 200 units of T7 RNA polymerase. All enzymes were supplied by Promega (Milan, Italy).

Oocytes were obtained from adult females of *Xenopus laevis*. The frogs were anaesthetized in 0.10% (w/v) MS222 (tricaine methansulfonate) solution in tap water and portions of the ovary were removed through an incision on the abdomen. The oocytes were treated with 1 mg/ml collagenase (Sigma Type IA) in ND96 calcium-free solution for at least 1 h at 18°C. After 24 h at 18°C in modified Barth's saline solution (MBS), the healthy looking oocytes were injected with 25 ng of cRNA in 50 nl of water, using a manual microinjection system (Drummond). The oocytes were then incubated at 18°C for 3–4 days in MBS before electrophysiological or transport studies. The experiments were carried out according to institutional and national ethical guidelines (permit nr. 05/12).

### Metal-sensitive fluorophore transport assay in *Dictyostelium* macropinosomes and in *Xenopus* oocytes, and confocal fluorescence imaging

For detecting calcein in macropinosomes, exponentially growing *Dictyostelium* cells expressing Nramp1(C)– or Nramp2(C)–GFP were incubated with 0.025 mM calcein (Sigma-Aldrich, St Louis, MO), diluted in growth medium, for 30 min under shaking; they were then washed, resuspended in 0.017 M Soerensen Na-K phosphate buffer pH 6.0 (Bozzaro and Roseman, 1983) and plated on 6×6 cm glass coverslips. For colocalization studies, cells were incubated with 1 mg/ml TRITC–dextran (Sigma-Aldrich) and 0.025 μM calcein in suspension. After 30 min, cells were washed in Soerensen phosphate buffer and plated on a 6×6 cm glass coverslip. Confocal series images were taken on an inverted Zeiss LSM510 microscope equipped with a 100× Neofluar 1.3 oil immersion objective, using a multi-track configuration as follows. Track 1 excitation was with a 543-nm band from a helium-neon laser line, emission collected with a 620 nm long-pass filter for TRITC. Track 2 excitation was with a 488-nm band from an argon-ion laser line and emission collected with a 510–525 nm bandpass filter for FITC (Peracino et al., 1998, 2010). The scanning interval time was reduced to 1.9 s.

For monitoring metal transport in *Dictyostelium* macropinosomes, cells were incubated with increasing amounts of divalent metals and 0.025 mM calcein in axenic growth medium for 30 min, washed in Soerensen phosphate buffer and incubated on glass coverslips. For continuous loading experiments, exponentially growing control or mutant cells were washed, resuspended in low fluorescence medium (LoFlo Medium supplemented with yeast extract, Formedium, Norfolk, UK) and plated on a 6×6 cm glass coverslip equipped with a 5-cm diameter Plexiglas ring. After gently adding calcein, chelated or not with FeCl_3_ or FeCl_2_, consecutive images of single cells were taken every 2.9 s on the confocal microscope.

For metal transport in *Xenopus* oocytes, control oocytes and oocytes transfected with cRNA encoding c-Nramp1, c-Nramp2 or rDMT1 were injected with a 50 nl drop of 0.025 mM calcein dissolved in intracellular solution (130 mM KCl, 4 mM NaCl, 1.6 mM MgCl_2_, 5 mM EGTA, 10 mM HEPES pH 7.6 and 5 mM glucose). The nominal volume of a 1.2-mm diameter oocyte is 1 µl; therefore, a 50-nl drop will be diluted 20 times. The exact dilution factor is, however, difficult to establish because not all the theoretical volume will necessarily be available for free diffusion (Bröer, 2010). Following calcein injection, the oocytes were placed in TMA solution at pH 5.5 containing or not divalent metals at a final concentration of 0.1 mM and observed at the confocal microscope equipped with a 5× Plan Neofluar 0.15 objective. Images of single oocytes were taken every 10 s for a total of 10 min, by using excitation at 488 nm and emission at 505–550 nm. For F:F0 quantification, the fluorescence intensity at time 0 (F0) and at subsequent times (F) was calculated in the entire area of the oocytes using ImageJ. Changes in fluorescence intensity in the entire oocyte or in selected areas were proportionally linear with time.

### Flow cytometry measurement of divalent metal transport in *Dictyostelium* cells

Wild-type or Nramp mutant *Dictyostelium* cells were incubated with 0.025 mM calcein and increasing amount of divalent metals for 30 min, washed in Soerensen phosphate buffer, placed on ice and then analyzed on a FACScan (Becton Dickinson & Co., San Jose, CA). Cells were identified based on forward and side scatter. FITC fluorescence was measured in the FL1 channel (excitation wavelength: 488 nm; emission: 530–540 nm), and the mean fluorescence was determined for at least 20,000 cells. Background cell autofluorescence was subtracted from every value. To quantify the calcein fluorescence recovery, the fluorescence mean values of samples in the presence of divalent metals were normalized to the maximal fluorescence values of corresponding samples in the absence of metals and expressed as the percentage of maximal fluorescence.

### Electrophysiology and data analysis

The two-electrode voltage-clamp technique (TEVC, Oocyte Clamp OC-725B, Warner Instruments, Hamden, CT) was used. Intracellular glass microelectrodes were filled with 3 M KCl and had tip resistances between 0.5–4 MΩ. Agar bridges (3% agar in 3 M KCl) connected the bath electrodes to the experimental chamber. The holding potential (Vh) was −40 mV. Recording was conducted at a fixed voltage. WinWCP version 4.4.6 (J. Dempster, University of Strathclyde, UK) or Clampex 10.2 (Molecular Devices) was used to run the experiments. Data were analyzed using Clampfit 10.2 (Molecular Devices), and figures were prepared with Origin Pro 8.0 (Microcal Software Inc., Northampton, MA).

Transport currents were determined by subtracting the records in the absence of a substrate from the corresponding ones in its presence.

### Radiochemical uptake assay

Uptake experiments with oocytes were performed 4 days after cRNA injection. The uptake solution contained the indicated concentration of ^55^FeCl_2_ and 100 mM NaCl, 1.8 mM KCl, 0.6 mM CaCl_2_, 0.6 mM MgCl_2_, 10 mM Mes or Hepes at pH 5.5, 6.5, 7.5 or 8.0. 1 mM ascorbic acid (freshly prepared) was added to maintain iron in the reduced form unless otherwise indicated.

Groups of 8–10 oocytes were incubated for 60 min in uptake solution, washed in ice-cold uptake solution devoid of FeCl_2_, dissolved in 10% SDS solution and counted in a liquid scintillation counter (Giovanola et al., 2012). Figures show mean±s.e.m. values for groups of 8–10 oocytes in three independent experiments. For statistical analysis, Student's *t*-test was applied.

## Supplementary Material

Supplementary Material
