# Supplementary material for: Dictyostelium Nramp1, which is structurally and functionally similar to mammalian DMT1 transporter, mediates phagosomal iron efflux
Source: J Cell Sci. 2015 Sep 1;128(17):3304–16. doi: 10.1242/jcs.173153 (PMC4582194; doi:10.1242/jcs.173153)
Supplement: Supplementary Material [file supp_128_17_3304__index.html]

Dictyostelium Nramp1, which is structurally and functionally similar to mammalian DMT1 transporter, mediates phagosomal iron efflux — Supplementary Material 

# *Dictyostelium* Nramp1, which is structurally and functionally similar to mammalian DMT1 transporter, mediates phagosomal iron efflux

## JCS173153 Supplementary Material

- Supplementary Material
