# Supplementary material for: Dictyostelium Nramp1, which is structurally and functionally similar to mammalian DMT1 transporter, mediates phagosomal iron efflux
Source: J Cell Sci. 2015 Sep 1;128(17):3304–16. doi: 10.1242/jcs.173153 (PMC4582194; doi:10.1242/jcs.173153)
Supplement: Supplementary Material [file supp_jcs.173153_JCS173153supp.pdf]

Supplementary material

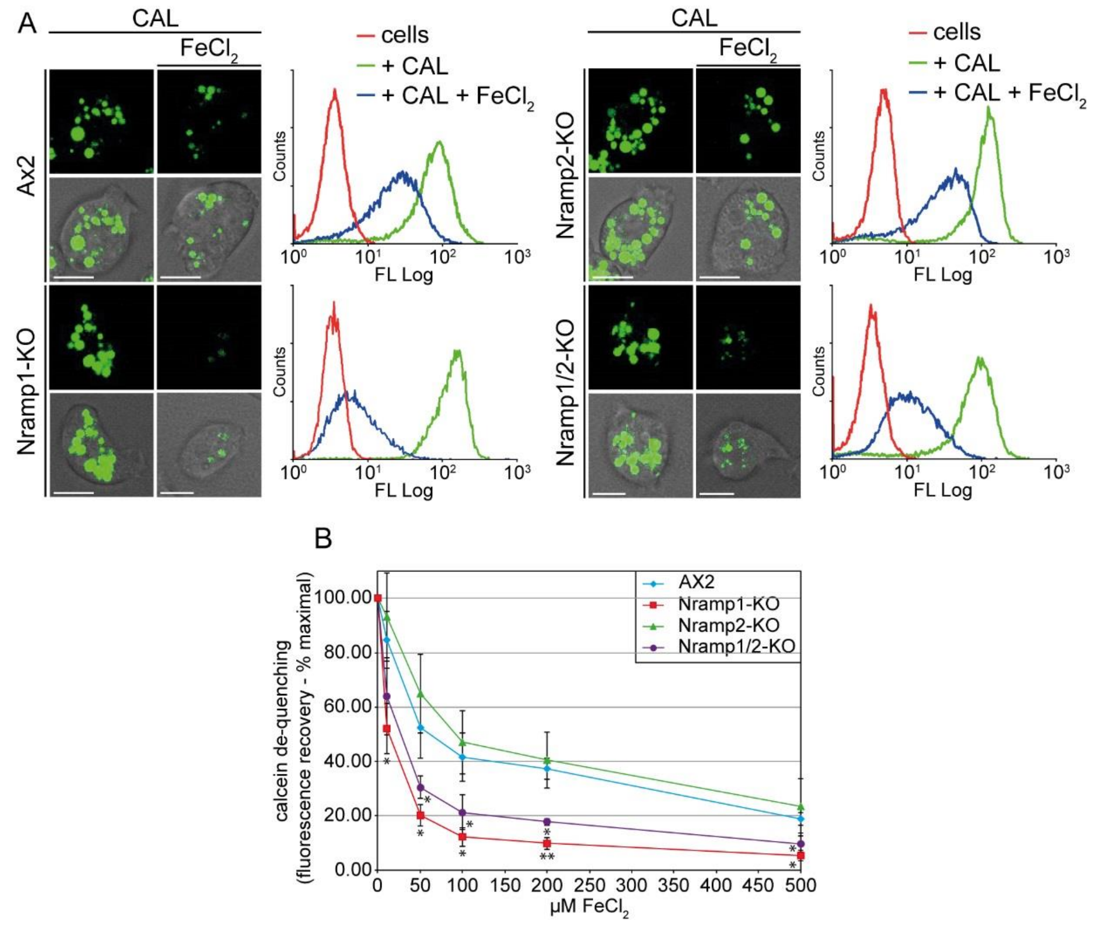

Figure S1. Recovery of iron-quenched calcein fluorescence in macropinosomes of AX2 and Nramp1 or Nramp2 knockout mutants.

(A-B) Cells of the wild type Ax2 of the indicated mutants were incubated with calcein in the presence or absence of 0.1 mM $FeCl_2$ for 30 min. At the end of the incubation, cells were washed, plated on coverslips and observed at the confocal microscope. In parallel, cell samples were analysed by fluorescence flow cytometry. Several macropinosomes containing calcein are visible in all cells in the absence of iron. In cells incubated with iron-quenched calcein, fluorescence recovery is evident in AX2 and Nramp2-null cells, no or very little recovery is observed in Nramp1- or Nramp1/Nramp2-null mutant. Cell flow cytometry data confirm the microscopy observations.

(C) Cells were incubated with calcein and increasing concentrations of $FeCl_2$ for 30 min, washed and analysed for calcein fluorescence in flow cytometer. The fluorescence intensity of the samples was expressed as % of maximal and plotted versus the initial concentration of iron. It is evident that even at non-saturating concentrations, i.e. when calcein is only partially quenched by iron, fluorescence recovery in macropinosomes is significantly lower in Nramp1- and Nramp1/Nramp2- null mutants than in AX2 or Nramp2-null mutant. *: $P < 0.05$; ** $P < 0.01$.

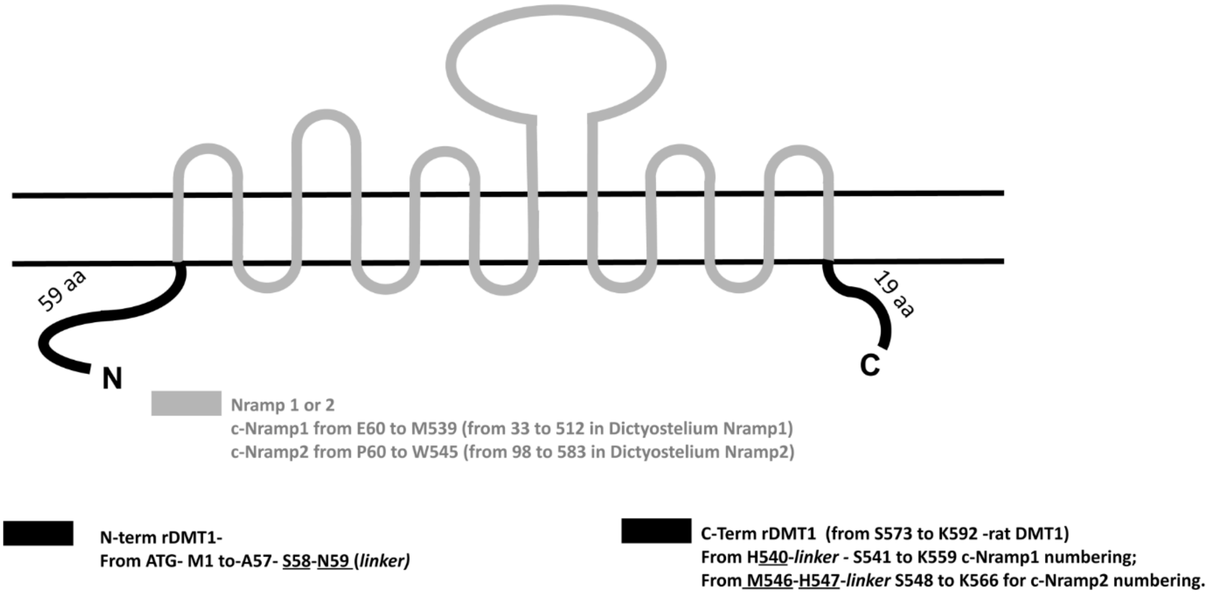

Figure S2. Scheme and construction of the chimeric proteins.

In grey the 12 TM domains of *Dictyostelium* protein Nramp1 or 2, in black the C- and N-terminus of rat DMT1 (NP037305.2). For each protein are reported the first and the last aminoacid and the aminoacids used as "linker" with the relative position in the original and chimeric protein. Chimeric cDNAs were prepared by joining together portion of cDNA coding for N- (60 amino acids) and C- terminus (19 amino acids) of rDMT1 (NP037305.2) in pSPORT1 and the main coding sequence for the twelve-transmembrane domains of *Dictyostelium nramp*1 or 2. In summary, to generate chimeric cDNAs, the restriction sites HpaI at the N-terminus (position +96 and +291 for *Dictyostelium nramp1* and *nramp2*, respectively) and NsiI at the C-terminus (position +1533 and +1749 for *nramp1* and *nramp2*, respectively) were inserted by PCR primer amplification of the central portion of cDNA coding for the proteins in pGEMT vector. Rat *dmt1* in pSPORT1 was instead mutagenized by site directed mutagenesis with overlapping primer to insert the Eco47III site (at position +171) and then the NsiI site (at position +1713). The final *c-nramp1* in pSPORT1 vector is coding for a protein with the first 59 amino acids of rat *dmt1* replacing the 32 residues at the N-terminus of *Dictyostelium nramp1,* which is the main coding sequence of the construct, where the 21 C-terminal amino acids are replaced by the last 19 amino acid of the rat protein. Instead, in the *c-nramp2* chimera, the same N-terminus and C-terminus of rat *dmt1* replace the first 97 and the last 46 residues of *Dictyostelium* Nramp2. Due to the peculiar AT rich sequence of Dictyostelium nramp genes, the following

conditions in the PCR protocols were used: DNA 50 ng, 1.25 ng of each primer, dNTPs 0,5mM, 3U of Pfu Taq, buffer in a total of 50 µl reaction. Thermocycling conditions: 1min of initial denaturation then for 25 cycles denaturation (95°C) 1 min, annealing (60°C) 1 min, extension (72°C) 3 min, followed by 5 min of extension at 72°C. The primer sequences were:

pSPORT_DMT1_Nsi_ fw 5'-gttgggcctgtcgttcctggactgtatgcattcggtaagcatct-3'

pSPORT_DMT1_Eco47III fw 5'-tcggccagcctcggtgccagcgctcctgcttacagcaac-3'

fw HpaI_Nramp1DD 5’-gcccGTTAACgaaaaccctttaataatagaaagtgg-3’

rev NsiI_Nramp1DD 5’-CACTGCAGAACCAATGCATagaattaaagttttcttgacccattG-3’

fw HpaI_Nramp2DD 5’-gcccGTTAACccatttcaagatagagatagtaatattgg-3’

rev NsiI_Nramp2DD 5’-CACTGCAGAACCAATGCATccataataataaacaacataaagcaattG-3’

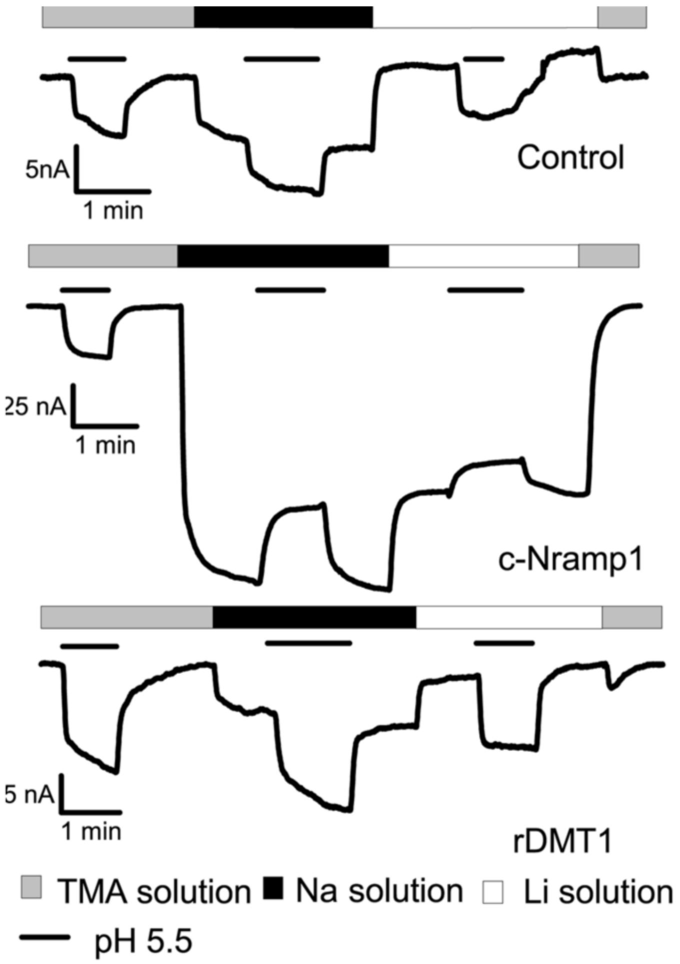

Figure S3. Slippage currents in c-Nramp1 and rDMT1-expressing oocytes.
Sodium and lithium leak currents were recorded at different pH in control, c-Nramp1 or rDMT1 expressing oocytes. Non transfected representative oocytes (*top*) or oocytes expressing c-Nramp1 (*middle*) or rDMT1 (*bottom*) were clamped at a constant voltage of -40 mV and perfused with the indicated TMA, sodium or lithium solution at 98 mM and pH 7.6 or pH 5.5 (*black straight line*) Currents were recorded as described in Material and Methods. Note the different scale for c-nramp1. For c-Nramp1, the current amplitude in sodium solution at pH 7.6 was of about -150 nA, and was reduced of about 50 nA when the pH was switched at 5.5, showing competition between sodium and protons for permeation through the protein. In lithium, the behaviour was similar, an uncoupled current of -110 nA was present at pH 7.6 and reduced to -90nA at pH 5.5. When the impermeant TMA solution was perfused, an inward current of about -30nA was recorded only at acidic pH, showing that in the absence of a permeating cation only slippage of protons occurs. The same solutions and pH conditions tested in oocytes expressing rDMT1 showed an inward small (10 to 15 nA) current at pH 5.5 independently of the perfused solution. This is due to protons entering the transporter even in the absence of divalent ion. In control oocytes, the acidic pH slightly depolarize the resting membrane potential. This is visualized in voltage clamp as an inward current of 2 to 5 nA. It is interesting that in both metal transporters at -40 mV sodium was more permeant than lithium even if a large slippage current that competes with protons is present only in *Dictyostelium* Nramp1. This behaviour in the presence of sodium ions is analogous to that reported for yeast Smf1p.
